# A Kidney Transplant Recipient on Prophylactic Eculizumab Presenting With Myalgia

**DOI:** 10.3389/ti.2022.10910

**Published:** 2022-12-08

**Authors:** Oshini Shivakumar, Rebekah Judge, Andrew Smith, Andreas Kousios

**Affiliations:** ^1^ West London Renal and Transplant Centre, Hammersmith Hospital, Imperial College Healthcare NHS Trust, London, United Kingdom; ^2^ North West London Pathology, Charing Cross Hospital, London, United Kingdom; ^3^ Department Immunology and Inflammation, Faculty of Medicine, Centre for Inflammatory Disease, Imperial College, London, United Kingdom

**Keywords:** Eculizumab, atypical hemolytic uremic syndrome, acute kidney injury, renal allograft, myalgia

## Case Report

A 50-year-old renal transplant recipient presented with 1 week history of myalgia and fever. His past medical history was remarkable for a deceased donor kidney transplant 16 months prior to presentation. The cause of renal disease leading to end stage kidney disease (ESKD) prior to his transplant was atypical haemolytic uraemic syndrome (aHUS) with thrombotic microangiopathy (TMA) on the native kidney biopsy (also had features of advanced interstitial fibrosis and tubular atrophy). Subsequent investigations confirmed heterozygous complement factor I mutation. His treatment included single-agent immunosuppression with tacrolimus and fortnightly prophylactic Eculizumab, since his transplant.

**FIGURE 1:**
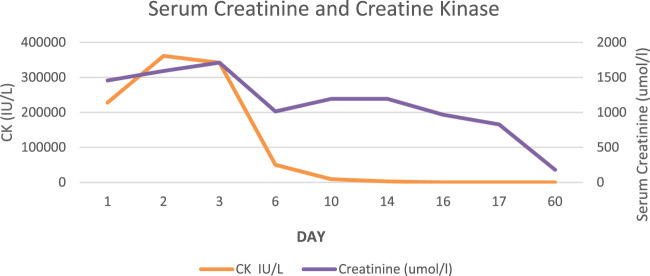
Serum creatinine (umol/l) and creatine kinase (IU/L).

**FIGURE 2:**
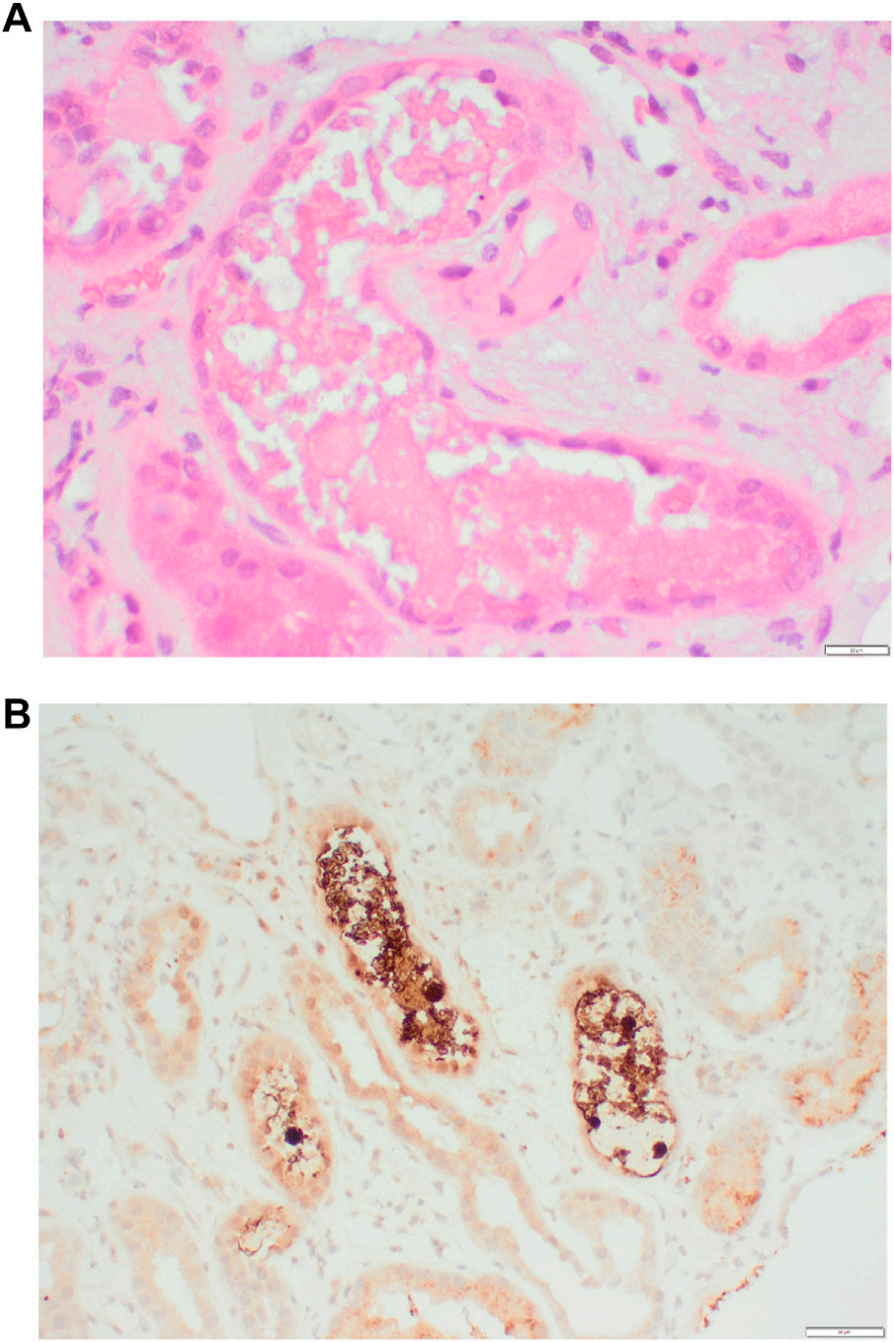
**(A)** Granular “stringy” and “beaded” cast suspicious for myoglobulin and **(B)** Positive immunohistochemistry (brown) in myoglobin casts.

**FIGURE 3:**
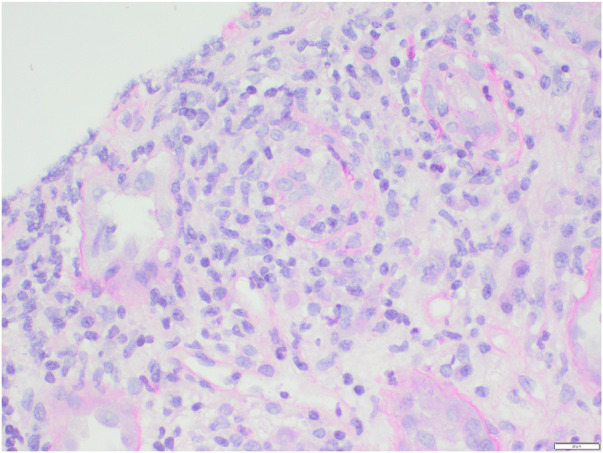
Tubulitis with between 5 and 10 intraepithelial lymphocytes per tubular cross section and discontinuity of the tubular basement membrane (t3).

On admission, he was normotensive and oligo-anuric with dark urine. A COVID-19 rapid test was positive and was confirmed with nasopharyngeal RT-PCR (no previous vaccination). He had missed one dose of Eculizumab. Laboratory tests revealed significant AKI with a creatinine of 1450 umol/l (baseline 180 umol/l), urea 41.1 mmol/l, LDH 12300U/L, CRP 264 mg/l (Figure 1). His haemoglobin, platelet count and haptoglobin levels were normal without fragments on a blood film and complement levels were normal. He was treated with intravenous fluids, broad spectrum antibiotics and dexamethasone according to the local protocol at the time. He did not respond to volume expansion and required intermittent haemodialysis. In view of the on-going need for dialysis more than 2 weeks following his admission a kidney graft biopsy was performed (Figures 2A,B, 3).

## Test Questions

(1) Which patients are recommended to have prophylactic Eculizumab prior to kidney transplant?
  (a) Isolated membrane co-factor (MCP) protein mutations
  (b) Persistently negative Factor H autoantibodies
  (c) Pathogenic variant in *CFH* or *CFI*
  (d) Pathogenic variant in a non-complement pathway gene, eg., DGKε
  (e) Previous graft loss due to allograft rejection
(2) Which maintenance immunosuppression agent should be avoided in patients at risk of recurrent aHUS?
  (a) Ciclosporin
  (b) Tacrolimus
  (c) Sirolimus
  (d) MMF
  (e) Azathioprine
(3) What is the histological finding from renal biopsy in Figure 2A,B?
  (a) Oxalate crystals
  (b) Myoglobin casts (rhabdomyolysis)
  (c) Cholesterol emboli
  (d) Acute Thrombotic microangiopathy
  (e) Rejection
(4) What is the most significant histological finding from renal biopsy in Figure 3?
  (a) Glomerulitis
  (b) Tubulitis
  (c) Peritubular capillaritis
  (d) Acute thrombotic microangiopathy
  (e) Intimal arteritis

## Data Availability

The original contributions presented in the study are included in the article/supplementary material, further inquiries can be directed to the corresponding author.

## Appendix

### Answers and Discussion

#### Question 1

**The correct answer is c.**

A typical HUS accounts for 10% presentations of HUS, with 70% of the patients have either a genetic or acquired defect in the regulation of the alternative pathway of complement activation. Around 50% of these patients either die or develop ESKD within 1 year of diagnosis. The risk of recurrence post transplantation is high (up to 60%) with poor graft outcomes. Eculizumab, a humanised Anti-C5 monoclonal antibody, has been shown to be effective for treatment of aHUS, in both native and kidney transplant patients. Current recommendations include the use of prophylactic Eculizumab in those individuals deemed medium or high risk of recurrence. These include patients with previous early aHUS recurrence, pathogenic variants in *CFH* or gene re-arrangements involving *CFH* and Factor H-related proteins, gain of function pathogenic variants in *CFB* or C3, pathogenic variant in *CFI* and those without identified mutation or with persistent low-titre anti-CFH antibody. Patients with the pathogenic variants of Membrane Cofactor Protein (MCP) have the lowest recurrence risk and no recurrence is seen in those with absent DGKε function (1).

#### Question 2

**The correct answer is c.**

Early use of mTOR inhibitors is an independent risk factor for the development of TMA (2). With regards to other immunosuppressive agents, there is conflicting evidence of their association with aHUS recurrence. Although, calcineurin inhibitors (CNIs), have been associated with *de novo* TMA, avoiding CNIs did not reduce the risk of recurrence of aHUS. Nonetheless, their use has the established advantage in reducing allograft rejection. Tacrolimus has a lower rate of post-transplant TMA incidence, hence is recommended as per transplant protocol published in 2021 by the National Renal Complement Therapeutics Centre (NRCTC) UK (3).

#### Question 3

**The correct answer is b.**

The figure demonstrates myglobin casts secondary to COVID-19 associated rhabdomyolysis. The patients’ CK on presentation was 350000 IU/l with anuric AKI and uraemia needing dialysis. Rhabdomyolysis refers to the lysis of striated muscle with glomerular filtration of myoglobin, which binds Tamm-Horsfall proteins leading to cast formation and distal tubular obstruction. The morphology of the myoglobin casts ranged from slightly brown granular casts by hematoxylin and eosin, to beaded globular casts that stained brightly fuchsinophilic with Masson trichrome and partially argyrophilic with silver methenamine. Calcium oxalate and cholesterol crystals dissolve during routine histological preparation. Calcium oxalate is seen as intratubular crystals of multicoloured birefringence under polarised light, with classic fan-shaped morphology. Cholesterol crystal emboli are usually defined by empty, biconvex and needle-shaped clefts, whereas in frozen sections, the crystals are birefringent under polarised light. Diagnostic features of TMA include fibrin thrombi within glomerular capillary loops in the acute stage and double contouring of glomerular basement membrane and intimal proliferation of arterioles in the chronic stage.

We have identified 38 case reports and series of COVID-19 associated rhabdomyolysis with detailed clinical data, including 54 individual cases, on MEDLINE. 80% (*n* = 43) were male, 18% (*n* = 10) female, and 2% (*n* = 1) unspecified. Median age was 54 (19–88) years. There was high burden of comorbidities, including Hypertension (41%, *n* = 22), pre-existing CKD (11%, *n* = 6), and Diabetes (19%, *n* = 10). 11% (*n* = 6) were taking statin. CK was elevated at presentation in 50% (*n* = 27), with a mean of 70,049 IU/L. CK peaked at presentation in 33% (*n* = 18) and peaked after presentation in 65% (*n* = 35). 54% (*n* = 29) of patients had AKI, with 69% (20/29) evident at presentation. 33% (*n* = 18) required haemofiltration and 3.7% (*n* = 2) remained dialysis-dependent after discharge. A large retrospective study of 1014 hospitalised patients with COVID-19 showed a 2.2% incidence of rhabdomyolysis. Peak CK levels >1000IU/L were an independent risk factor for in-hospital death (HR = 6.46, 95%CI:3.02–13.86) (4).

Rhabdomyolysis should be considered in all patients with COVID-19, presenting with AKI. A biopsy could be pursued if alternative diagnoses are considered or renal function is not improving. Whether rhabdomyolysis is a marker of severe disease; existing as part of a multi-system inflammatory disorder or a driver of mortality in itself, remains unclear.

#### Question 4

**The correct answer is b.**

Figure 3 shows lymphocytic infiltration of the tubules (tubulitis). Our patient was diagnosed with COVID-19 associated rhabdomyolysis and concomitant T cellmediated rejection (Type 1B). Without a biopsy the diagnosis of TCMR would have been missed. Early treatment with steroids and subsequent addition of MMF (4 weeks post COVID-19), improved renal function back to his previous baseline (Figure 1) and the patient is dialysis independent.
